# Interpretable Machine Learning for Risk Stratification of Hippocampal Atrophy in Alzheimer’s Disease Using CSF Erythrocyte Load and Clinical Data

**DOI:** 10.3390/biomedicines13112689

**Published:** 2025-10-31

**Authors:** Rafail C. Christodoulou, Georgios Vamvouras, Platon S. Papageorgiou, Maria Daniela Sarquis, Vasileia Petrou, Ludwing Rivera, Celimar Morales, Gipsany Rivera, Sokratis G. Papageorgiou, Evros Vassiliou

**Affiliations:** 1 Department of Radiology, Stanford University School of Medicine, Stanford, CA 94305, USA; 2 Department of Mechanical Engineering, National Technical University of Athens, 15772 Zografou, Greece; gvamvouras@mail.ntua.gr; 3 2nd Department of Orthopaedic Surgery and Traumatology, Aghia Sophia Pediatric General Hospital, Thivon 3 Street, 15772 Athens, Greece; 4 Department of Medicine, Universidad de Carabobo, Valencia 2001, Venezuela; mdsarquis58@gmail.com; 5 Department of Medicine, University of Ioannina, 45110 Ioannina, Greece; md07010@uoi.gr; 6 Department of Medicine, American University of Antigua, Jabberwock Road, Osbourn 999152, Antigua and Barbuda; ludwingr@auamed.net (L.R.); celimarm@auamed.net (C.M.); gipsanyr@auamed.net (G.R.); 7 1st Department of Neurology, Medical School, National and Kapodistrian University of Athens, Eginition Hospital, 15772 Athens, Greece; 8 Department of Biological Sciences, Kean University, Union, NJ 07083, USA

**Keywords:** Alzheimer’s disease, hippocampal atrophy, machine learning, CSF erythrocytes, mean arterial pressure, progression, clinical data, interpretable AI, risk prediction, follow-up

## Abstract

**Background/Objectives:** Hippocampal atrophy indicates Alzheimer’s disease (AD) progression and guides follow-up and trial enrichment. Identifying high-risk patients is crucial for optimizing care, but accessible, interpretable machine-learning models (ML) are limited. We developed an explainable ML model using clinical data and CSF erythrocyte load (CTRED) to classify adults with AD as high- or low-risk based on hippocampal volume decline. **Methods:** Included ADNI participants with ≥2 MRIs, baseline lumbar puncture, and vital signs within 6 months of MRI (n = 26). The outcome was the Annual Percentage Change (APC) in hippocampal volume, classified as low or high risk. Predictors were standardized; models included SVM, logistic regression, and Ridge Classifier, tuned and tested on a set (n = 6). Thresholds were based on out-of-fold predictions under a 10–90% positive rate. Explainability used PFI and SHAP for per-patient contributions. **Results:** All models gave identical classifications, but discrimination varied: Ridge AUC = 1.00, logistic = 0.889, and SVM = 0.667. PFI highlighted MAPres and sex as main signals; CTRED contributed, and age had a minor impact. **Conclusions:** The explainable ML model with clinical data and CTRED can stratify AD patients by hippocampal atrophy risk, aiding follow-up and vascular assessment planning rather than treatment decisions. Validation in larger cohorts is needed. This is the first ML study to use CSF erythrocyte load to predict hippocampal atrophy risk in AD.

## 1. Introduction

Alzheimer’s disease (AD) is a progressive neurodegenerative disorder and the most common cause of dementia, representing a major global health and socioeconomic challenge. It is characterized by widespread brain atrophy, particularly within the hippocampus and medial temporal lobes, leading to progressive memory and executive function impairment. Over seven million Americans are currently living with AD, and this number is projected to exceed 13 million by 2050 [1]. Patients with AD show significant impairment in memory and executive functioning. It is characterized by progressive neurodegeneration with prominent medial temporal lobe involvement [2,3]. Among temporal imaging markers, hippocampal atrophy is one of the most sensitive and specific disease severity and progression indicators [4]. Hippocampal atrophy is widely used in research and clinical practice for diagnosis, monitoring, and clinical trial enrolment. Large-scale studies have shown that longitudinal MRI-derived hippocampal measurements capture clinically meaningful trajectories of the AD spectrum [5,6,7]. Patients who convert from MCI to AD showed 1.3% higher hippocampal atrophy compared to stable MCI [8]. Notably, an autopsy validated work demonstrated that MRI-derived hippocampal volume is a sensitive and specific index of AD neuropathology. Hippocampal volume correlates with neuritic plaque burden and neuritic tangle stage postmortem, a surrogate of disease neuropathology stage [9].

Despite this progress, a significant unmet need persists in determining which individuals are most at risk for rapid hippocampal volume decline. That stratification could help structure the visit timeline, therapeutic timeline (anti-amyloid or vascular risk management strategies), and stratification for clinical trials [10]. While previous prognostic models have used multimodal biomarkers like genetics, PET, MRI, and clinical data or imaging deep learning, many of these methods are still hard to implement in everyday clinical practice because of their cost and complex Pipelines [11,12]. In addition, many high-performance ML systems are also “black boxes,” limiting transparency and clinical trust. Efforts toward quantitatively interpretable architectures aim to bridge this gap by exposing feature contributions [13,14]. Recent studies confirm that although many prediction models report high performance, most remain unvalidated externally, and their complexity challenges translation to routine practice [15,16]. Therefore, this underscores the benefit of intrinsically interpretable models in high-stakes domains like neurology [17]. Given the large patient volume and the high costs and delays associated with complex tests like PET scans and genetic analysis, there is an urgent demand for interpretable standard ML frameworks that produce biologically plausible predictions from routinely available data. Beyond the standard amyloid and tau measures, vascular and demographic features influence neurodegeneration [18]. Age and sex are the strong non-modifiable risk factors, while blood pressure has been reported to accelerate atrophy through various hemodynamic mechanisms. Women’s brain atrophy rates were roughly 1.5% higher than men’s, while younger individuals exhibited atrophy rates about 1% higher than older ones due to higher tau levels [11,18]. The role of blood pressure (BP) in hippocampal atrophy is highly complex. High BP in midlife has been associated with increased brain atrophy later in life. Studies on older individuals have shown the reverse, where low BP was associated with enhanced neurodegeneration [19]. Furthermore, high blood pressure is often associated with white matter lesions (WMLs), contributing to brain atrophy [19,20]. These variables are readily available, which justifies their inclusion in prognostic models.

Recent evidence also implicates erythrocyte load in cerebrospinal fluid (CSF) as relevant to structural neurodegeneration. In our prior study, elevated CSF erythrocytes were associated with greater hippocampal atrophy in AD patients, suggesting that CSF erythrocyte load (CTRED) may carry prognostic information for neurodegenerative diseases [21,22]. This finding supports the increasing research linking erythrocyte-derived and iron-related processes to AD pathophysiology. Iron metabolism and erythrocyte balance disruptions, such as elevated hemoglobin, ferritin, and heme oxygenase-1 activity, are connected to higher amyloid levels, hippocampal shrinking, and worsening cognitive function [23,24,25]. Mechanistically, erythrocytes’ oxidative stress and redox imbalance can impair oxygen transport and trigger peroxidative damage in the cerebral microvasculature [26,27,28]. Dysregulated iron handling contributes to ferroptosis, microvascular fragility, and accumulation of paramagnetic iron species, which can be detected using susceptibility-based MRI techniques [29]. Elevated CSF iron species have also been linked to dementia risk in population studies, supporting that erythrocyte-related biomarkers reflect ongoing vascular and metabolic injury rather than procedural contamination [24]. Metabolic and antioxidant abnormalities in circulating erythrocytes can impair cerebral oxygen delivery and promote downstream neurodegeneration, positioning RBC-related measures as potential risk indicators for AD [30]. These findings position CTRED as a biologically plausible and reliable surrogate marker of neurovascular integrity, oxidative stress, and hippocampal vulnerability in AD.

Machine learning (ML) is becoming increasingly prevalent in AD prognosis. However, interpretable models and small footprints (penalized linear methods and SVMs) are more feasible to validate and adopt clinically than data-intensive models [31,32]. Explainability methods, such as Permutation Feature Importance (PFI) and SHAP, reveal the importance of each variable in risk estimation [33].

This study aimed to determine if a simple, interpretable machine-learning (ML) model can predict hippocampal volume decline in AD using routine clinical and laboratory data. The model integrates cerebrospinal fluid erythrocyte load (CTRED), MAPres, age, and sex to classify patients as high or low risk for ongoing hippocampal atrophy. We hypothesized that vascular and hematologic markers, particularly MAPres and CTRED, would independently improve predictions of structural decline, emphasizing the link between vascular dysregulation, microvascular health, and neurodegeneration in AD. A comprehensive literature review revealed no previous ML studies using a CSF erythrocyte load to predict hippocampal volume loss in AD. Most research on CSF erythrocytes concentrates on their influence on other biomarkers, not structural atrophy prediction. Thus, we aim to develop and assess an interpretable ML model combining CTRED, MAPres, age, and sex to categorize AD patients by their risk of hippocampal degeneration [34,35].

## 2. Materials and Methods

### 2.1. Study Design and Objective

This study employed a reproducible, interpretable machine-learning (ML) pipeline to stratify Alzheimer’s disease (AD) patients by risk of hippocampal volume decline using routine clinical and cerebrospinal fluid (CSF) variables. The approach was guided by the previous literature demonstrating that small-footprint linear models can provide transparent and clinically plausible predictions in neurodegenerative research [14,31,32]. In particular, we focused on standardized, low-dimensional predictors such as mean arterial pressure (MAPres), CSF erythrocyte load (CTRED), age, and sex, which have been independently associated with hippocampal atrophy and vascular dysregulation in AD [10,18,19,20,22]. A summary of the cohort characteristics is shown in Table 1.

Table 2 provides an overview of the main methodological stages, linking each to prior methodological precedents. All steps, from data extraction to model explainability, were implemented using scikit-learn (1.4.2) and Optuna (4.4.0), emphasizing reproducibility and interpretability over algorithmic complexity.

### 2.2. Data Source and Ethics

Data were obtained from the ADNI dataset (http://adni.loni.usc.edu, accessed on 9 March 2025). The ADNI project was launched in 2003 as a public–private partnership with the primary goal of testing whether clinical, imaging, genetic, and biochemical biomarkers can be combined to measure the progression of mild cognitive impairment (MCI) and early Alzheimer’s Disease (AD). All participants gave written informed consent for data collection and sharing during enrolment. The study protocols and consent forms were approved by each participating institution’s institutional review boards (IRBs).

### 2.3. Eligibility and Cohort Construction

Inclusion in the study required at least two visits, each with an MRI taken, along with a spinal tap and recorded vital signs, at a temporal proximity to the MRIs of 6 months at most.

### 2.4. Outcome Definition (Annual Percentage Change, APC)

Risk of hippocampal volume decline is quantified as the per-subject Annual Percentage Change (APC) of normalized average hippocampal volume, which is defined as

(1) $$hipp_{norm}=\frac{Left\;Hippocampus+Right\;Hippocampus}{2*total\;brain\;volume}$$

(2) $$APC=\frac{last\;hipp_{norm}\;measurement-first\;hipp_{norm}\;measurement}{first\;hipp_{norm}\;measurement}*\frac{100\%}{years\;between\;scans}$$

**Figure 1:**
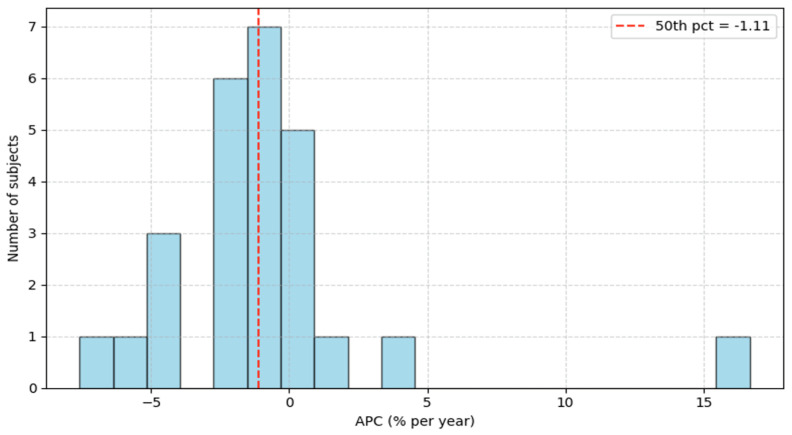
Annual Percentage Change histogram.

The left and right hippocampus volumes and the total brain volume were measured using FreeSurfer, as described in [22]. The available subjects were 26 and separated into two groups, labeled as low and high risk, based on the APC value. Specifically, subjects above the 50th percentile were labeled as high risk, whereas those below the 50th percentile were labeled as low risk, as seen in Figure 1. The corresponding APC value at the 50th percentile is −1.11%, and low-risk subjects are denoted by label 0, contrary to higher-risk subjects, denoted by label 1.

### 2.5. Predictor Variables

Baseline predictors were age, sex, cerebrospinal fluid erythrocyte load (CTRED), and mean arterial pressure (MAPres), measured at the earliest qualifying (baseline) visit within ≤6 months of the baseline MRI. The modeling objective was to classify subjects into low vs. high risk of hippocampal volume decline. Data were split into training (n = 20) and test (n = 6) sets.

### 2.6. Preprocessing

Continuous predictors (CTRED, age, and MAPres) were z-scaled within scikit-learn Pipelines using means/SDs that only fit on the training split and were applied unchanged to the test split to prevent information leakage. Sex was encoded as a binary indicator (male = 0, female = 1). MAPres was computed from baseline systolic and diastolic blood pressures as defined in Equation (3), using measurements recorded within ≤6 months of the baseline MRI (per eligibility criteria). No imputation was required for the final analytic cohort; participants with missing predictor values were excluded during cohort construction. No outlier removal or winsorization was performed. Feature-importance rankings remained qualitatively consistent after alternative CTRED weighting schemes, reinforcing that our findings are unlikely to stem solely from procedural artifacts. This reliability is supported by ADNI’s standardized CSF collection procedures, which enforce strict sample-handling protocols to minimize erythrocyte contamination (e.g., atraumatic LP techniques and pre-analytical controls). All transformations (scaling and encoding) were encapsulated in the model Pipelines used during cross-validation and final fitting. Class balance was not altered at the preprocessing stage; model-level class_weight settings were used where applicable (see Section 2.7). Outcome derivations (hippocampal normalization and APC) are detailed in Section 2.4.

(3) $$MAPres\;=\;\frac{3\cdot DBP\;+\;2\cdot SBP}{3}$$

### 2.7. Models

We evaluated three linear classifiers, two of which comprise a soft-voting ensemble. All models were implemented in scikit-learn within Pipelines that applied z-scaling to continuous inputs (see Section 2.6).

(i) Support Vector Machine (SVM)

A linear SVM was built using SVC (kernel = “linear”) inside a Pipeline with StandardScaler. We set class_weight = “balanced” and enabled Platt scaling (probability = True) to obtain calibrated probabilities for downstream ensembling.

(ii) Logistic Regression

A penalized logistic regression classifier was implemented in a Pipeline with StandardScaler. Coefficients were estimated by maximum likelihood under L1 or L2 penalty, with solver chosen among liblinear or saga during tuning. Class weights were explored as either “none” or “balanced” in the search space.

(iii) Ridge Classifier

A Ridge Classifier was implemented in a Pipeline with StandardScaler, using class_weight = “balanced”. Because Ridge Classifier exposes a decision function rather than calibrated probabilities, it was not included in the probability-averaging ensemble; the regularization strength α was tuned in Section 2.8.

(iv) Soft-Voting Ensemble

The ensemble combined the SVM and logistic regression by averaging their predicted probabilities for the positive class to produce a single ensemble probability. A single decision threshold was selected on the training set under a 10–90% predicted-positive-rate constraint when no candidate satisfied the constraint, Youden’s J (TPR + TNR − 1) was a fallback (Section 2.9).

### 2.8. Hyperparameter Optimization

Hyperparameter tuning was performed with Optuna (Tree-structured Parzen Estimator, TPE), running 500 trials per model on the training split only. During tuning, performance was estimated by stratified k-fold cross-validation on the training data; the model with the best mean CV score was refit on the full training set. Out-of-fold (OOF) probabilities from the training split were retained for decision-threshold selection (Section 2.9).

- Linear SVM

Search space (log-uniform): $C\in \left[10^{-3},\;10^{3}\right]$

CV objective: F1-score (mean over folds) 

Implementation details: SVC (kernel = “linear”, class_weight = “balanced”, probability = True) inside a Pipeline with StandardScaler.

- Logistic Regression

Search space: penalty ∈ {L1, L2}; solver ∈ {liblinear, saga}; $C\in \left[10^{-3},\;10^{3}\right]\;$(log-uniform); max_iter ∈ [500, 5000]; class_weight ∈ {None, balanced}.

CV objective: F1-score (mean over folds) 

CV folds: k capped by the minority-class count (min 2, max 5).

- Ridge Classifier

Search space (log-uniform): $\alpha \in \left[10^{-3},\;10^{3}\right]$

CV objective: F1-score (mean over folds).

Note: RidgeClassifier outputs a decision function (not calibrated probabilities) and was therefore excluded from probability averaging in the ensemble.

To ensure reproducibility, fixed random seeds were used where applicable, and all preprocessing (standardization) was embedded within the model Pipelines invoked by Optuna so that each CV fold applied identical transformations learned inside the fold.

### 2.9. Decision Thresholding

For logistic regression, hyperparameters were tuned by CV ROC-AUC. Out-of-fold probabilities were used to sweep thresholds t ∈ [0.01, 0.99], selecting the one that minimized the right-hand side of (Equation (4)) under a 10–90% positive-rate constraint; fallback was Youden’s J, as defined in Equation (5). This fixed threshold was applied to the refit model for test evaluation.

(4) $$score=-\sqrt{{FP}^{2}+{FN}^{2}}$$

(5) J=sensitivity+specificity−1 (equivalently TPR−FPR)

For the linear SVM, probabilities were obtained with Platt scaling (probability = True). Results are reported using the default 0.5 cutoff, and no custom thresholding was applied.

For the soft-voting ensemble, model probabilities were averaged, and the threshold was tuned during training to maximize accuracy under the same 10–90% positive-rate constraint, again with Youden’s J as fallback.

### 2.10. Evaluation and Metrics

Model performance was assessed on the held-out test set (n = 6) using threshold-independent and threshold-dependent metrics.

Primary metrics.

- ROC–AUC (threshold-independent) computed from predicted probabilities (Logistic, SVM, Ensemble) or decision scores (Ridge).
- Accuracy, Precision, Recall, and F1-score computed at a single fixed decision threshold selected on the training split as described in Section 2.9. Metrics are reported per class (low-risk = 0; high-risk = 1) together with the confusion matrix.

Training-split reporting.

For transparency in a small-N setting, we additionally report training-split performance for the ensemble (AUC and accuracy) while emphasizing the held-out test results as the primary estimate of generalization.

Implementation.

All metrics were computed with scikit-learn confusion matrices, and class-wise metrics reflect the fixed thresholds determined on the training split.

### 2.11. Software and Reproducibility

Analyses were conducted in Python (v3.12.3) using scikit-learn (v1.4.2) for model development, Optuna (v4.4.0) for hyperparameter optimization, and PFI for explainability. All continuous-feature standardization and categorical encodings were encapsulated in scikit-learn Pipelines to avoid data leakage (train-only fitting applied to test data). Random seeds were fixed where applicable to enhance reproducibility.

## 3. Results

### 3.1. Cohort and Outcome Summary

A total of 26 ADNI participants met eligibility criteria (≥2 visits with MRI; baseline lumbar puncture and vital signs within ≤6 months of the baseline MRI). The outcome was the Annual Percentage Change (APC) in normalized average hippocampal volume. The APC distribution is shown in [Figure 1]. The cohort median was −1.11%, which defined the risk labels: subjects below the median were labeled low risk (label = 0), and those above the median were labeled high risk (label = 1). This median split yielded two groups of comparable sizes.

Baseline predictors CSF erythrocyte load (CTRED), age, sex (male = 0, female = 1), and mean arterial pressure (MAPres) were taken from the baseline visit, with MAPres computed from SBP/DBP Data were randomly partitioned into training (n = 20) and test (n = 6) sets; all preprocessing and tuning steps were confined to the training split before a single evaluation on the held-out test set.

### 3.2. Model Performance

#### 3.2.1. Linear SVM

**Figure 2:**
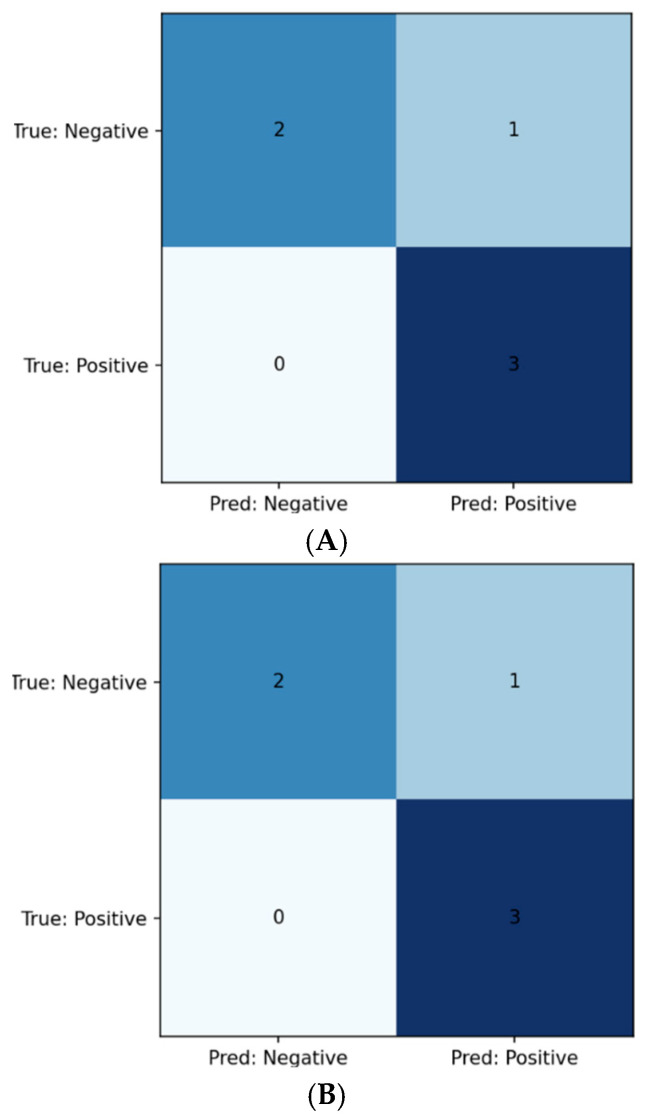

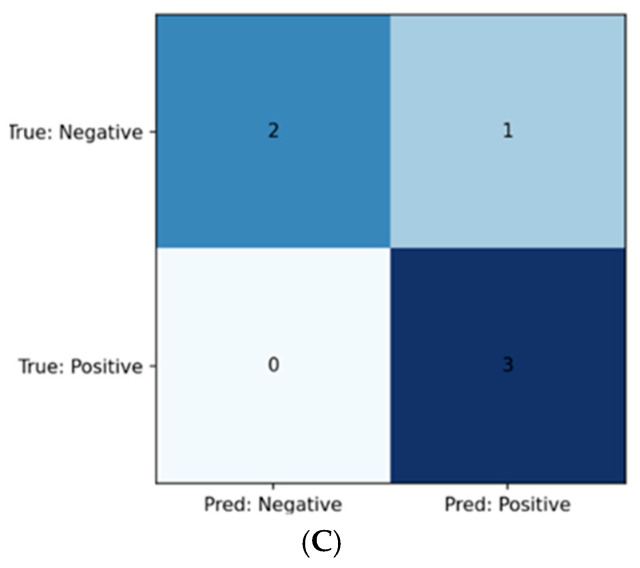
(**A**) Confusion matrix for SVM model; (**B**) Confusion matrix for logistic regression model; (**C**) Confusion matrix for Ridge model.

**Figure 3:**
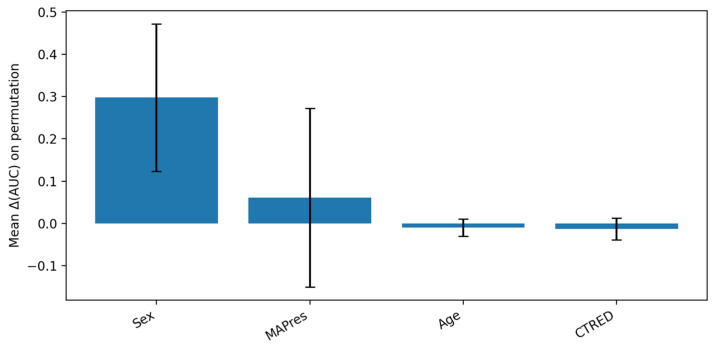
PFI analysis for SVM model. The bar plot shows a relatively high mean of sex contribution to AUC disruption upon feature permutation (tall blue column), which is accompanied by a high variance (long black whiskers), due to limited sample folds. Meanwhile, AUC appears to depend less on MAPres, but the high variance that is introduced by the different dependency level in each fold renders the result ambiguous. Additionally, the slight variance associated with age and CTRED shows that the model did not depend on those features as much as on sex and age; however, their importance should not be downplayed.

After tuning the regularization parameter C, the linear SVM achieved the following test-set metrics (Table 3): Class 0 Precision 1.000, Recall 0.667, F1 0.800; Class 1 Precision 0.750, Recall 1.000, F1 0.857. The ROC–AUC was 0.667. The confusion matrix is shown in Figure 2A. (Permutation-based feature rankings are summarized in Section 3.3; PFI shown in Figure 3).

#### 3.2.2. Penalized Logistic Regression

With hyperparameters selected as in Table 4, the logistic regression classifier yielded identical class-wise test metrics to the SVM (Table 5): Class 0 Precision 1.000, Recall 0.667, F1 0.800 Class 1 Precision 0.750, Recall 1.000, F1 0.857. The ROC–AUC was 0.889. The confusion matrix appears in Figure 2B.

#### 3.2.3. Ridge Classifier

Following α optimization, the Ridge classifier produced the same class-wise test metrics as the regression model, seen in (Table 6): Class 0 Precision 1.000, Recall 0.667, F1 0.800; Class 1 Precision 0.750, Recall 1.000, F1 0.857. Using the continuous decision scores, the ROC–AUC was 1.000. The confusion matrix is shown in Figure 2C.

#### 3.2.4. Soft-Voting Ensemble

Averaging calibrated probabilities from the SVM and logistic models (Section 2.7), the ensemble achieved on the training split: AUC 0.910, accuracy 0.850 (Table 7). On the held-out test set, performance was AUC 0.889, accuracy 0.833, with class-wise metrics matching the single models (Table 8).

In summary, all models (including the ensemble) produced identical test-set classifications (i.e., the same confusion matrix), while AUC differentiated discriminative ability across decision thresholds: Ridge > Logistic/Ensemble > SVM. Given the small test sample (n = 6), these estimates should be interpreted cautiously; nonetheless, the agreement in threshold-dependent metrics across models suggests that the signal is stable.

### 3.3. Explainability

Permutation Feature Importance (PFI). On the held-out test set, we estimated global importance by repeatedly shuffling one predictor at a time and re-scoring the model; the average drop in performance (compared to the unshuffled baseline) quantified that predictor’s importance. For the linear SVM, PFI identified sex and MAPres as the most influential predictors, followed by CTRED and age (Figure 3). This ranking aligned with standardized coefficients from the linear models, supporting a consistent linear signal across methods.

PFI measures model reliance rather than effect direction; thus, we do not infer whether higher or lower values increase risk. In this context, MAPres is interpreted as a marker of cerebrovascular status or dysregulation, and CTRED as an RBC or vascular integrity signal, with interpretation considering possible procedural influences (e.g., traumatic tap).

**Figure 4:**
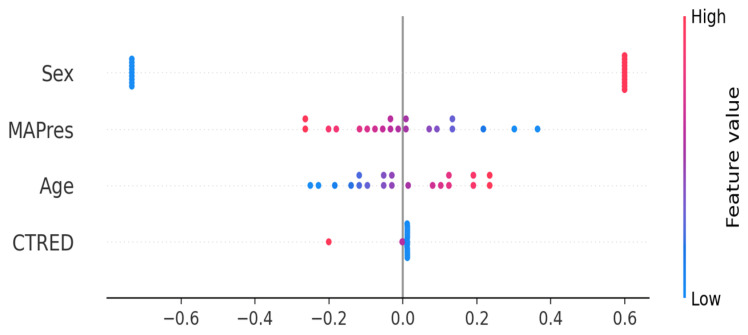
SHAP analysis for the Ridge classifier. Blue denotes low feature value (e.g., low age) and red indicates high feature value (e.g., higher age). Female subjects (in blue) show a high positive association with increased prediction risk, unlike male subjects, who show a high negative association with risk. High MAPres is associated with lower hippocampal volume reduction, whereas higher age is associated with a higher prediction risk.

To better understand directionality and individual effects, we used SHAP analysis on the Ridge classifier (see Figure 4). SHAP values showed that female sex increased prediction risk, while male sex was linked to a decrease. High MAPres correlated with a lower predicted risk of hippocampal volume reduction, whereas higher age was associated with an increased risk. Notably, CTRED was not among the major predictors of this study; however this does not mean it is redundant ML models tend to focus more on strong predictors like MAPres and sex when available, leading to their dominion over the rest, like age and CTRED. These results offer additional insights: PFI identified which predictors the models focused on most, while SHAP explained how specific feature values affected individual prediction levels.

Overall, the classification of hippocampal atrophy risk was consistent across all models, with similar predicted labels across methods. Among linear approaches, the Ridge classifier achieved the highest discrimination (AUC = 1.00), followed by logistic regression and the soft-voting ensemble (AUC = 0.889), while the SVM showed lower discrimination (AUC = 0.667). Feature-importance analyses consistently identified MAPres and sex as dominant predictors, with CTRED and age contributing secondary effects. These findings collectively support the reliability and biological plausibility of the interpretable ML pipeline.

## 4. Discussion

Given the unmet need to stratify patients with a high risk of hippocampal decline using accessible clinical data, we employed a simple approach. Previous methods used proteomics, clinical data, and imaging features to predict AD progression or specifically focus on hippocampal atrophy [10,36,37]. We used a compact, readily available feature set CSF CTRED, age, sex, and MAPres, to develop interpretable classifiers that differentiate AD patients into high or low risk of future hippocampal volume decline. On the held-out test set (n = 6), all models produced identical class assignments (i.e., the same confusion matrix and class-specific Precision/Recall/F1), while threshold-independent discrimination (AUC) separated them: Ridge = 1.000, logistic regression = 0.889, soft-voting ensemble = 0.889, and linear SVM = 0.667. Ensemble performance on the training split (AUC = 0.910; accuracy = 0.850) and the test split (AUC = 0.889, accuracy = 0.833) further supports the consistency of the learned signal across thresholds. Permutation Feature Importance confirmed a consistent pattern: MAPres and sex had the most significant influence on risk classification, CTRED added positive discriminative value across its range, and age contributed a more negligible, monotonic effect.

These findings demonstrate that, using available information, a small, transparent modeling approach can generate coherent and clinically plausible risk stratification of hippocampal atrophy. However, estimates, especially the perfect Ridge AUC, should be interpreted cautiously due to the small sizes of the training and test sets.

The feature-importance pattern revealed by PFI and SHAP indicated MAPres and sex as dominant contributors, with CTRED and age showing secondary yet consistent effects of smaller magnitude. This feature pattern aligns biologically with hippocampal degeneration in AD. MAPres is a significant contributor to classification, indicating its importance in predicting atrophy risk. The relationship between BP and AD is highly complex and controversial [38]. Prior studies suggest that elevated blood pressure and hypotension can be associated with hippocampal atrophy and cognitive decline through different mechanisms [39]. Higher baseline BP was linked to greater subsequent hippocampal atrophy, while reduced systolic blood pressure led to faster declines in MMSE scores. Notably, no relationship was found between normotensive patients and cognitive scores [40]. High BP damages the cerebral blood vessels’ structure and function, which can harm white matter regions critical for cognitive function. Studies suggest that cumulative BP effects on cerebral vasculature over time may worsen the situation, though evidence that antihypertensive treatment improves cognition or slows atrophy in AD remains inconclusive [34]. Few studies have examined the relationship of low BP with AD, but population-based data indicate that low BP predisposes individuals to AD and dementia. Importantly, patients with AD can have lower BP as a consequence of degeneration in the autonomic nervous system [35].

The sex effect can be a valuable variable in predicting atrophy in AD. Longitudinal studies have previously shown sex differences in brain atrophy rates and cognitive and functional decline. Ardekani (2016) et al. showed that in MIRIAD AD patients, the hippocampal atrophy rates in women were significantly faster compared to men, with 6.61% versus 4.31% (*p* = 0.008) [18]. This difference is attributed to biological, genetic, or even social factors linked to gender, such as occupation and education level. Hormonal profiles, especially estrogens, play a central role. Estrogens promote glucose uptake, which is the brain’s primary energy source. As estrogen levels decline during menopause, this reduces glucose uptake by neurons. Consequently, neurons must switch to auxiliary sources like lipids, including white matter. Damage to white matter worsens the risk and promotes AD progression [41,42]. Our SHAP analysis of the Ridge classifier supported this literature, showing that female sex contributed positively to prediction risk, while male sex showed a protective (negative) contribution.

CTRED emerged as a predictor of risk classification, indicating that cerebrospinal erythrocyte signal points to hippocampal atrophy risk. In previous work, higher CTRED was linked to greater hippocampal atrophy in the AD cohort [22]. This finding supports previous ADNI studies showing that CSF hemoglobin and ferritin levels are indicators of erythrocyte load predicting cognitive decline and faster conversion from MCI to AD, even after accounting for potential RBC contamination [25,43]. Several mechanisms might explain this link.

**Figure 5:**
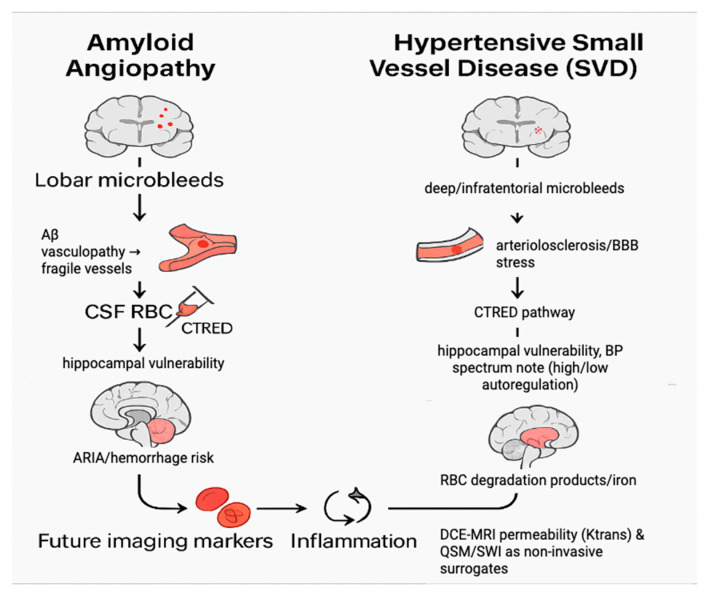
Mechanistic pathways linking CTRED to hippocampal atrophy.

CTRED may serve as a surrogate for microvascular fragility or subtle hemorrhagic events, especially in the context of amyloid-related angiopathy in AD. Amyloid deposits weaken vessel walls, making them prone to bleeding [44]. Alternatively, with studies reporting hypertension up to 51%, microhemorrhages can result from vessel leakage (Figure 5) [45]. When RBCs degrade, their products might induce neuroinflammation and neuronal stress [46]. However, because CTRED can also be influenced by procedural factors (e.g., traumatic tap), it is advisable to interpret it cautiously if proper procedural protocols have not been followed. Clinical context and procedural details should be considered. Although the impact of age was modest, aligning with ADNI data showing slower atrophy with age in AD/MCI and faster decline in younger patients, it reduces age-related variance in older cohorts [10]. Multivariable analyses suggest that once pathology, like Aβ, is accounted for, age adds minimal extra information about hippocampal atrophy, especially due to range restrictions in limited age groups [47]. Consistent with this, SHAP analysis showed that higher age continued to positively influence the predicted risk in our model, reflecting residual variance in our specific cohort rather than a strong independent biological factor. The model indicates that hippocampal decline results from multiple factors, such as vascular strain (MAPres), sex-related biology, RBC-related processes, and, to a lesser extent, age. Since these variables are often measured at baseline, an interpretable classifier that includes them can provide meaningful, pathophysiologically grounded risk predictions. 

On the left, amyloid angiopathy is shown as a pathway of lobar microbleeds and β-amyloid vasculopathy, which weaken vessel walls and allow red blood cells to enter the CSF, thereby increasing hippocampal atrophy risk. On the right, hypertensive small-vessel disease (SVD) is depicted as a pathway involving deep or infratentorial microbleeds, chronic blood–brain barrier stress, and impaired autoregulation, which also elevates CTRED and promotes hippocampal degeneration. In both mechanisms, degradation of red blood cells and iron deposition contribute to inflammation and oxidative stress. Future work may leverage non-invasive surrogates such as DCE-MRI permeability mapping (Ktrans) and QSM/SWI-based iron measures to approximate CTRED without lumbar puncture.

The interpretability analysis used PFI and SHAP. Both identified MAPres and sex as key signals, with CTRED and age being less influential, supporting face validity that the model behaves plausibly for clinicians. While face validity boosts confidence that results are not artifacts, it does not replace external validation. We avoid directional claims, interpreting MAPres as a cerebrovascular marker rather than a strict risk indicator, aligned with literature on pressure states and brain vulnerability. CTRED is an RBC/vascular integrity signal with biological and maybe procedural influences. SHAP complemented PFI by clarifying directionality, showing that female sex contributed positively to risk, high MAPres was associated with lower predicted risk, and higher age was associated with increased risk, which aligns with the prior literature. However, not only is AD a multifactorial process, with the relationship between MAPres or age being extremely complex in directionality, but also the feature importance of the variables can vary between ML models.

This study presents a proof-of-concept modeling framework to explore whether routinely obtainable variables, such as CTRED, mean arterial pressure, can predict outcomes. MAPres, age, and sex can jointly predict the risk of hippocampal volume decline in AD. The framework demonstrates how these parameters, when available from baseline MRI, LP, and clinical records, may be integrated into an interpretable model producing binary risk classifications (high versus low) and feature-importance estimates derived from PFI. Standard quality-control steps, such as reviewing LP reports, verifying blood-pressure measurements, and assessing MRI volumetric reliability, are assumed within the analytic pipeline.

CTRED shows a substantial contribution to the model’s predictions, and traumatic tap appears unlikely based on procedural notes; the signal may reflect aspects of vascular integrity rather than contamination. In such circumstances, corresponding imaging patterns observed on microbleed-sensitive MRI sequences (e.g., SWI or T2*) could help contextualize the result. Lobar-predominant microbleeds are typically associated with amyloid-related angiopathy, while deep or infratentorial patterns align more with hypertensive small-vessel disease, acknowledging that mixed profiles frequently occur [48,49]. Where an amyloid-dominant mechanism is suspected, the finding may conceptually align with established literature linking amyloid pathology to microvascular fragility and hemorrhagic risk, particularly in individuals receiving antithrombotic therapy [44,50,51]. Conversely, a hypertensive profile is consistent with studies emphasizing the impact of chronic blood-pressure dysregulation on hippocampal integrity [52].

When MAPres emerges as a major explanatory variable, the direction of its coefficient offers interpretive context, suggesting whether elevated or reduced pressures dominate within the modeled relationship. Low-pressure associations could, for instance, correspond to effects of medication, dehydration, autonomic dysfunction, or endocrine disturbances (e.g., adrenal or thyroid disorders) [53]. Age and sex operate as fixed demographic covariates that primarily modulate risk communication rather than representing direct intervention targets.

**Figure 6:**
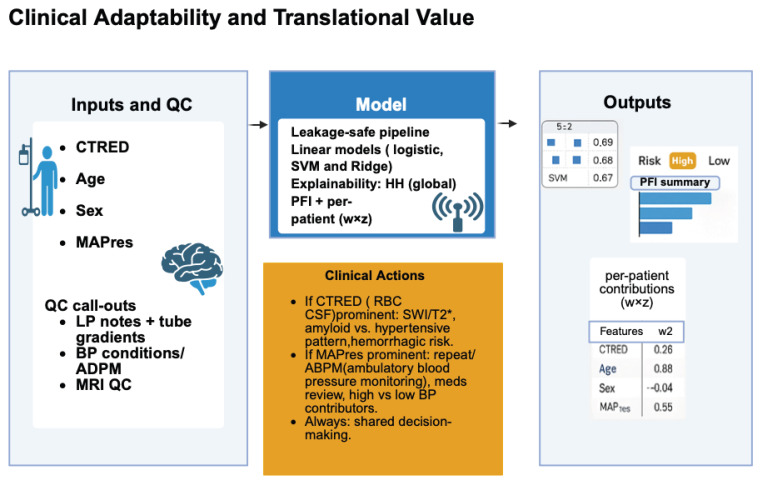
Illustrates a workflow for risk stratification of hippocampal volume decline in Alzheimer’s disease using accessible measures. Inputs include cerebrospinal fluid erythrocyte load (CTRED), age, sex, and mean arterial pressure (MAPres). Quality control verifies lumbar puncture reports, blood pressure, and MRI quality for volumetric analysis. These are processed through a leakage-safe machine-learning pipeline with linear models (logistic regression, Ridge, and SVM) to classify risk as high or low. Interpretability is maintained globally via Permutation Feature Importance (PFI) and at the patient level with SHAP values. Outputs support clinicians: CTRED highlights microbleed-related MRI and hemorrhagic risk, while MAPres emphasizes blood pressure assessment and monitoring.

Overall, this framework (Figure 6) should be interpreted as an early-stage, hypothesis-generating prototype illustrating the potential integration of hematologic and vascular measures into explainable ML models of structural neurodegeneration.

This study has some limitations. Firstly, it is a proof-of-concept study with a small group of AD patients (n = 26), so its findings may not be broadly applicable and should be considered preliminary until further validation is conducted. CTRED might not be accessible for all patients because it requires LP measurement, which can be complicated by blood contamination. While the LP protocols are carried out per strict ADNI guidelines, a small degree of contamination cannot be completely ruled out. Additionally, the model needs volumetric measurements as input, necessitating dedicated volumetric software for MRI scans. This software can be costly, particularly for smaller medical facility centers.

Future research should aim at developing a non-invasive CTRED surrogate. First, CTRED should be correlated with QSM (regional susceptibility/iron) and SWI/T2* microbleed burden and patterns to determine an imaging cut-point that approximates the current CTRED threshold. Meanwhile, a classifier to predict high versus low CTRED based on imaging and routine variables (QSM/SWI features, MAPres, P-tau, Aβ) and assess whether replacing actual CTRED with its predicted value maintains risk classification accuracy after calibration and external validation in larger cohorts. To explore vascular mechanisms, DCE-MRI can be used to calculate Ktrans, a standard index of BBB leakiness, to evaluate its added prognostic value and potential interactions with MAPres (e.g., subgroups defined by pressure signal and BBB effects) status.

## 5. Conclusions

This study presents a proof-of-concept interpretable machine-learning framework integrating CTRED, MAPres, age, and sex to stratify adults with AD by risk of hippocampal volume decline. Despite the limited sample, the models showed consistent classification performance, with Ridge regression achieving the highest discrimination (AUC = 1.00). Feature-importance analyses highlighted MAPres and sex as dominant predictors, supporting biological plausibility. These findings suggest that routine vascular and hematologic measures may carry prognostic value for neurodegeneration. While preliminary and requiring external validation, this work underscores the potential of transparent, small-footprint models to illuminate vascular contributions to AD pathology and to inform personalized follow-up strategies once confirmed in larger cohorts.

## Figures and Tables

**Table 1:** Descriptive statistics.

Characteristic	Values
Age mean (SD)	73.8 (7.6)
Sex	
Male	11
Female	15
APC yearly % change (mean, SD)	−0.9 (4.3)
CTRED (mean, SD)	121.5 (526.8)
MAPres (mean, SD)	92.3 (8.7)

**Table 2:** Main methodological stages.

Data acquisition	ADNI dataset with MRI, CSF, and vital signs	[10,21,22]
Outcome derivation	Annual Percentage Change in hippocampal volume	[5,6,7,8,9]
Predictor selection	CTRED, MAPres, age, and sex (all clinically accessible biomarkers)	[18,19,20,22,23]
Modeling	Linear SVM, logistic regression, Ridge classifier, and ensembling	[31,32,33]
Explainability	Permutation Feature Importance (PFI) and SHAP values	[17,33]

**Table 3:** Class-wise metrics on the SVM model.

	Precision	Recall	F1-Score
**Class 0**	1.000	0.667	0.800
**Class 1**	0.750	1.000	0.857

**Table 4:** Logistic regression model hyperparameters.

Hyperparameter	Explored Values
Trials	500
Solver	Libinear or saga
Penalty	L1 or L2
C value	[10^−3^, 10^3^] (log-uniform)
Maximum iterations	[500, 5000]
Class weight	None or balanced

**Table 5:** Class-wise metrics on the logistic regression model.

	Precision	Recall	F1-Score
**Class 0**	1.000	0.667	0.800
**Class 1**	0.750	1.000	0.857

**Table 6:** Class-wise Metrics on the Ridge Classifier.

	Precision	Recall	F1-Score
**Class 0**	1.000	0.667	0.800
**Class 1**	0.750	1.000	0.857

**Table 7:** Training set class-wise metrics on the ensemble model.

	Precision	Recall	F1-Score
**Class 0**	0.889	0.800	0.842
**Class 1**	0.818	0.900	0.857

**Table 8:** Test set class-wise metrics on the ensemble model.

	Precision	Recall	F1-Score
**Class 0**	1.000	0.667	0.800
**Class 1**	0.750	1.000	0.857

## Data Availability

The data presented in this study are available on request from the Alzheimer’s Disease Neuroimaging Initiative (ADNI) database: http://adni.loni.usc.edu, accessed on 9 March 2025. Access to the data requires registration and compliance with ADNI’s data use agreement. The authors did not generate any new datasets in this study.

## Abbreviations

AD	Alzheimer’s Disease
ADNI	Alzheimer’s Disease Neuroimaging Initiative
AI	Artificial Intelligence
APC	Annual Percentage Change
AUC	Area Under the Curve
BBB	Blood–Brain Barrier
BP	Blood Pressure
CC BY	Creative Commons Attribution
CI	Confidence Interval
CSF	Cerebrospinal Fluid
CTRED	Cerebrospinal Fluid Total Red Blood Cell (Erythrocyte) Load
DBP	Diastolic Blood Pressure
DCE-MRI	Dynamic Contrast-Enhanced Magnetic Resonance Imaging
DOI	Digital Object Identifier
F1	F1-score (Harmonic Mean of Precision and Recall)
IRB	Institutional Review Board
LP	Lumbar Puncture
MAPres	Mean Arterial Pressure
MCI	Mild Cognitive Impairment
ML	Machine Learning
MRI	Magnetic Resonance Imaging
n	Sample Size
OOF	Out-of-Fold
PFI	Permutation Feature Importance
P-tau	Phosphorylated Tau
QSM	Quantitative Susceptibility Mapping
RBC	Red Blood Cell
ROC	Receiver Operating Characteristic
SBP	Systolic Blood Pressure
SHAP	Shapley Additive Explanations
SVM	Support Vector Machine
SWI	Susceptibility-Weighted Imaging
T2*	T2-star Weighted Imaging
WML	White Matter Lesion
